# Comparative transcriptome analysis between inbred lines and hybrids provides molecular insights into K^+^ content heterosis of tobacco (*Nicotiana tabacum* L.)

**DOI:** 10.3389/fpls.2022.940787

**Published:** 2022-08-05

**Authors:** Zejun Mo, Wen Luo, Kai Pi, Lili Duan, Pingsong Wang, Yuzhou Ke, Shuaibo Zeng, Rongli Jia, Ting Liang, Ying Huang, Renxiang Liu

**Affiliations:** ^1^College of Agriculture, Guizhou University, Guiyang, China; ^2^Key Laboratory of Tobacco Quality in Guizhou Province, Guiyang, China; ^3^College of Tobacco, Guizhou University, Guiyang, China

**Keywords:** potassium, heterosis, transcriptomics, *Nicotiana tabacum*, quality

## Abstract

Potassium (K^+^) is essential for crop growth. Increasing the K^+^ content can often directly promote the improvement of crop yield and quality. Heterosis plays an important role in genetic improvement and leads to genetic gains. We found that the K^+^ content of tobacco showed significant heterosis, which is highly significant for cultivating tobacco varieties with high K^+^ content. However, the mechanism by which K^+^ content heterosis occurs in tobacco leaves is not clear. In this study, a comprehensive comparative transcriptome sequencing analysis of root samples from the hybrid G70 × GDH11 and its parental inbred lines G70 and GDH11 was performed to elucidate the importance of the root uptake capacity of K^+^ in the formation of heterosis. The results showed that 29.53% and 60.49% of the differentially expressed genes (DEGs) exhibited dominant and over-dominant expression patterns, respectively. These non-additive upregulated DEGs were significantly enriched in GO terms, such as metal ion transport and reaction, ion balance and homeostasis, ion channel activity, root meristem growth, and regulation of root hairs. The KEGG annotation results indicated that these genes were mainly involved in the pathways such as energy metabolism, carbohydrate formation, amino acid metabolism, and signal transduction. Further analysis showed that probable potassium transporter 17 (*NtKT17*) and potassium transporter 5-like (*NtKT5*), associated with potassium ion absorption, glutamate receptor 2.2-like and glutamate receptor 2.8-like, associated with ion channel activity, LOC107782957, protein detoxification 42-like, and probable glutamate carboxypeptidase 2, associated with root configuration, showed a significantly higher expression in the hybrids. These results indicated that the over-dominant expression pattern of DEGs played a key role in the heterosis of K^+^ content in tobacco leaves, and the overexpression of the genes related to K^+^ uptake, transport, and root development in hybrids helped to improve the K^+^ content of plants, thus showing the phenomenon of heterosis.

## Introduction

The new “green revolution” includes crop genetic improvement, healthy plant production, and efficient utilization of water and fertilizer (Beddington, 2010). Among them, the genetic improvement of crops ensures sustainable agricultural development through the cultivation of new varieties and the production of genetic gains (Wan, 2018). For the genetic improvement of crops, heterosis plays an important role in breeding desirable varieties and has led to remarkable achievements (Wu et al., 2021). Since it can greatly improve the production and quality of animals and plants, it significantly benefits society and the economy (Zhang et al., 2020), and also plays an important role in solving issues regarding global food security (Li et al., 2016).

Heterosis is a common biological phenomenon in which the hybrid offspring have better phenotypic traits than their parents (Hochholdinger and Baldauf, 2018). In China, it was found that the hybridization between horses and donkeys produced stronger mules (Yu et al., 2021). However, the study of plant heterosis began in Europe. In the mid-18th century, Kolreute performed tobacco hybridization between different species to cultivate early maturing tobacco hybrids with excellent qualities (Murad et al., 2002). Dominance (Davenport, 1908), overdominance (East, 1936), and epistasis (Williams, 1959) can explain heterosis partially, and their effects have been studied to some extent. Crop heterosis is not only different among different crops and traits, but the same trait of the same crop may be different due to different hybrid combinations, hybridization methods, and the cultivation environment (Schnable and Springer, 2013). Therefore, the mechanism of heterosis formation lacks a general explanation; specifically, heteropolyploid heterosis is relatively under-studied (Tian et al., 2018).

Tobacco (*Nicotiana tabacum* L.) is a special economic crop that is widely cultivated and has the characteristics of a short growth cycle, clear genetic background, and easy genetic transformation. It is also a model plant to study and elucidate various biological mechanisms (Xu et al., 2020; Zhang et al., 2022). Potassium ion (K^+^) is a macroelement required for plant growth and affects the yield, quality, and stress resistance of plants by participating in activities such as protein synthesis, cell osmotic regulation, photosynthesis, stomatal movement, and enzyme activation (Pettigrew, 2008; Ragel et al., 2019). Tobacco is a potassium-loving crop, and potassium can not only significantly improve the color, combustion power, and firepower of tobacco leaves but also increase their flexibility and softness (Wang et al., 2022). Therefore, high potassium content in tobacco leaves increases the yield and quality of the leaves.

Tobacco directly absorbs K^+^ from the soil through roots and subsequently accumulates K^+^ in the leaves. Potassium ion is transported to leaves through the xylem due to the expression of potassium channel genes and potassium transporter genes (Sano et al., 2009). The potassium absorption capacity of tobacco roots is closely related to the physiological characteristics (root active absorption area, root activity, ATPase activity, etc.) and root morphology (Li and Li, 2001; Li and Ma, 2003), and there are also considerable differences in the capacity to absorb K^+^ among different genotypes (Chen et al., 2010). Various genes related to K^+^ absorption and transport have been isolated and cloned. These genes play various roles in different stages of life (Chai et al., 2019) and include influx potassium channel genes: *AKT1* (Hirsch et al., 1998), *KAT1* (Sottocornola et al., 2008), *KC1* (Reintanz et al., 2002), etc.; outflow potassium channel genes: *SKOR* (Gaymard et al., 1998); and potassium transporter genes: *KUP1* (Santa-María et al., 2018), *HAK* (Greiner et al., 2011), *TPK1* (Gobert et al., 2007), etc. The mining of these key genes that regulate K^+^ absorption and transport is important for the directional improvement of potassium content in plants.

The potassium content of tobacco leaves increases in the topping period. After topping, the potassium content decreases rapidly, resulting in the low potassium content of mature tobacco leaves (Dai et al., 2009). Studies on improving tobacco quality focus on the strategies to effectively enhance the potassium content and select tobacco varieties with a high potassium content. In the early stages of research, we found that the heterosis of potassium content in tobacco leaves was prominent, and through heterosis utilization breeding, we obtained the hybrids Guiyan 202 and Guiyan 4 with high potassium content in tobacco leaves (Liu et al., 2006b, 2016). However, the mechanism of development of potassium content heterosis in tobacco leaves is unclear. Therefore, to determine the molecular components that might act on potassium heterosis, we performed transcriptome analysis of root samples of hybrids and their parents of the model plant common tobacco (*N. tabacum* L.) to elucidate the underlying mechanism of root potassium uptake capacity in the formation of potassium heterosis.

## Materials and methods

### Plant materials and planting

The tobacco hybrid F_1_ (G70 × GDH11) and two parental inbred lines (G70 and GDH11) selected for this study were provided by the Guizhou Key Laboratory of Tobacco Quality Research. We ensured that the collection of plant material and experimental research and field studies on plants complied with relevant institutional, national, and international guidelines and legislation. The seeds were sown in a special substrate for flue-cured tobacco and raised by floating the seedlings in a greenhouse. The seedlings were grown there till they had five true leaves, and then they were transplanted to the field. The field experiment was conducted in the tobacco scientific research and experimental base of Guizhou University in 2021, with three replicates and a row plant spacing of 110 cm × 55 cm. All plants were topped after 50% of the center flowers of the plants were open.

Samples were collected every 10 days between 60 and 90 days after transplantation. Three tobacco plants with the same growth performance were randomly selected from each plot, and their young fibrous roots and root tips were sampled and mixed together. The samples were first washed with clean water and then with PBS. Next, they were placed in separate sterilized centrifuge tubes, frozen with liquid nitrogen, and stored in a low-temperature refrigerator at −80°C at the earliest. The leaf samples were killed at 105°C for 30 min, then turned to 75°C to dry, ground into powder, bagged, and sealed for storage.

### Determination of K^+^ content and analysis of heterosis

To determine the content of K^+^ in leaves, 0.5 g of the sample was soaked in 0.5 mol/L of dilute hydrochloric acid and filtered. Then, the K^+^ content was determined using a flame spectrophotometer (Munns et al., 2010). The K^+^ content was calculated according to the following formula:


$$K^{+}\%=\frac{C\times V}{G\times 10^{6}}\times 100$$

Here, C indicates the K^+^ concentration (ppm) obtained from the standard curve; V indicates the volume of the liquid to be measured; G indicates the dry smoke sample weight (g); 10^6^ is the weight conversion coefficient.

The values of over high-parent heterosis (OPH), mid-parent heterosis (MPH), and below low-parent heterosis (BPH) were calculated according to the following formulae: $\mathrm{OPH}\;\left(\%\right)=\left(\frac{F1-\mathrm{HP}}{\mathrm{HP}}\right)\times 100$ , $\mathrm{MPH}\;\left(\%\right)=\left(\frac{F1-\mathrm{MP}}{\mathrm{MP}}\right)\times 100$ , $\mathrm{BPH}\;\left(\%\right)=\left(\frac{F1-\mathrm{LP}}{\mathrm{LP}}\right)\times 100$ , where F_1_ represents the first hybrid generation, HP represents the high-value parent, MP represents the average parent value $\left(\frac{\mathrm{parent}1+\mathrm{parent}2}{2}\right)$ , and LP represents the low-value parent.

### RNA isolation and library preparation

The biological samples of 70 days after transplanting were used as materials for RNA sequencing analysis. Total RNA from the root of nine samples was extracted using the TRIzol reagent following the manufacturer’s instructions (OE Biotech Co., Ltd., Item No.: T105096). The purity and quantity of RNA were evaluated using the NanoDrop 2000 spectrophotometer (Thermo Scientific, United States). The integrity of the RNA was assessed using the Agilent 2,100 Bioanalyzer (Agilent Technologies, Santa Clara, CA, United States). Then, the libraries were constructed using TruSeq Stranded mRNA LT Sample Prep Kit (Illumina, San Diego, CA, United States) following the manufacturer’s instructions.

### RNA sequencing and analysis of differentially expressed genes

The libraries were sequenced on an Illumina HiSeq X Ten platform, and 150 bp paired-end reads were generated. An average of 8.67 million reads was obtained per sample. Raw data (raw reads) in the fastq format were initially processed using Trimmomatic (Bolger et al., 2014), and the low-quality reads were removed to obtain clean reads. Then, about 8.60 million clean reads for each sample were retained for subsequent analyses.

The clean reads were mapped to the tobacco genome^1^ using HISAT2 (Kim et al., 2015). The Fragments Per Kilobase of exon model per million mapped fragments (FPKM; Roberts et al., 2011) of each gene was calculated using Cufflinks (Trapnell et al., 2011), and the read counts of each gene were obtained using the HTSeq-count tool (Anders et al., 2015). Differential expression analysis was performed using the DESeq2 (Anders and Huber, 2012); the value of *p* < 0.05 and foldchange >2 or foldchange <0.5 were set as the threshold for significant differential expression. Hierarchical cluster analysis of differentially expressed genes (DEGs) was performed to determine the expression pattern of genes in different groups and samples. The GO enrichment and KEGG pathway enrichment analyses of the DEGs were performed using the R software, based on the hypergeometric distribution.

### Real-time fluorescence quantitative PCR

To confirm the level of gene expression, based on the initial results of RNA sequencing, six genes were randomly selected for real-time fluorescence quantitative PCR (RT-qPCR) experiments. The experiments were performed using the SYBR Premix Ex Taq kit (Takara) and the Applied Biosystems 7500 Real-Time PCR system (Life Technologies Corporation, Beverly, MA, United States). The genes and corresponding primers used for the qPCR test are listed in Supplementary File 4. To calculate the relative expression level of each gene, the 2^−ΔΔCt^ method was used (Livak and Schmittgen, 2001).

### Statistical analyses

Duncan’s new multiple range tests were conducted to analyze the variation in the K^+^ content (*p* < 0.05) using the SPSS (version 16.0) software. Use Origin 2018 (95_64) and Adobe Illustrator CS6 for figure drawing.

## Results

### The performance of K^+^ content and its heterosis in tobacco leaves

**Figure 1:**
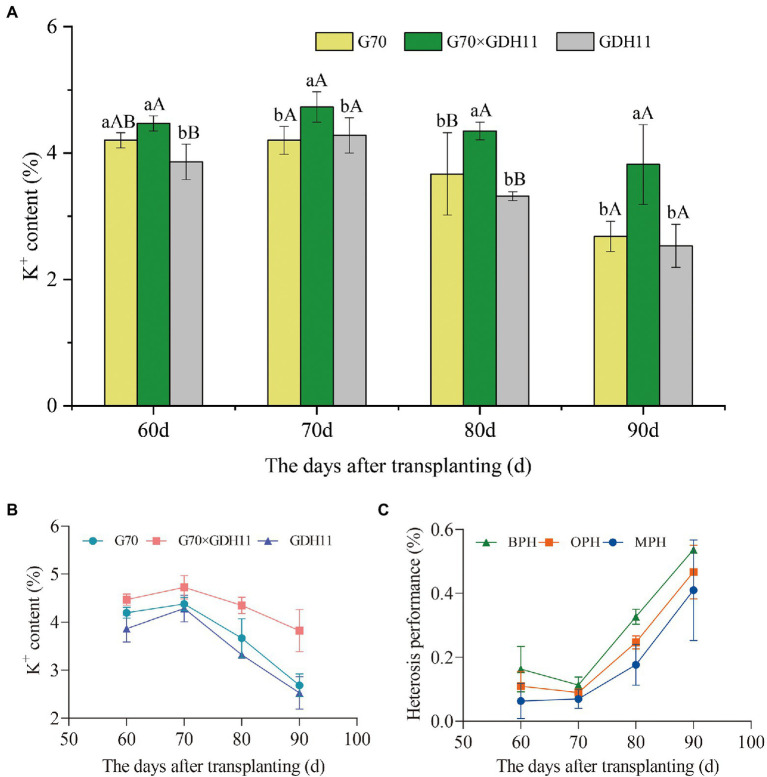
Potassium content of tobacco hybrids and their parents. **(A)** The K^+^ content of the hybrid G70 × GDH11 and its parents G70 and GDH11. **(B)** The changes in the K^+^ content in hybrid F_1_ and its parents between 60 and 90  days after transplantation. **(C)** The heterosis performance values of the hybrid G70 × GDH11. The average value of three biological replicates per material was used for mapping. Error bars represent significant differences among the three replicates. Significant differences in the K^+^ content at *p* < 0.05 and *p* < 0.01 were determined using Duncan’s new multiple range tests. The lowercase alphabets represent a significant difference (*p* < 0.05), while the uppercase alphabets represent a highly significant difference (*p* < 0.01).

To determine the difference in the K^+^ content between the hybrids and their parents in different growth periods, we took samples every 10 days between 60 and 90 days after transplantation and evaluated the K^+^ content in parents and their hybrids (Supplementary File 1). In the four growth stages studied, the K^+^ content of the hybrid G70 × GDH11 was significantly higher than that of the female parent G70 and the male parent GDH11 (Figure 1A). Between 60 and 90 days after transplantation, the K^+^ content of the three tested plant samples first increased and then decreased, and the shift (increase to decrease) in the K^+^ content in the plants occurred 70 days after transplantation (Figure 1B). Moreover, 70 days after transplantation, the K^+^ content in the two-parent samples decreased significantly more than that in the hybrid. Subsequently, we investigated the field agronomic traits of the hybrid and their parents (Supplementary Table S1), and found that the hybrid G70 × GDH11 performed significantly better over the two parents, indicating that the hybrids with strong K^+^ content advantage had more robust stalks, longer and wider leaves, and larger biomass weight. These results showed that the hybrid had a stronger K^+^ absorption capacity and a higher ability to retain K^+^ in the leaves.

The heterosis performance of G70 × GDH11 in different periods showed that K^+^ heterosis increased rapidly 70 days after transplantation (Figure 1C). Therefore, we took the plant material 70 days after transplantation as the experimental sample to investigate the key role of the absorptive capacity of tobacco roots in the formation of K^+^ content heterosis. Ninety days after transplantation, the OPH, MPH, and BPH of G70 × GDH11 were more than 40%, indicating that the K^+^ content of tobacco was higher in the hybrid offspring and heterosis breeding might produce hybrid plants with significantly higher K^+^ content than that in either parent plant.

### Differences in transcriptome expression between the roots of parents and hybrids

Using the hybrid G70 × GDH11 and its parents G70 and GDH11, RNA sequencing was performed using IlluminaHiSeq2000 to obtain a comprehensive reference transcriptome of nine tobacco root samples. The sequencing results were subjected to quality control analysis and sequence alignment. Information on the quality control analysis of the sequencing data is presented in Table 1, and the results of the comparison between the original data and the reference genome after quality control are presented in Table 2. With an average error rate of 0.02% across all samples, Q20 > 98%, Q30 > 94%, and GC content >42%, the clean sequences accounted for more than 99% of the total sequences (Table 1). The total sequence numbers and the results of genome mapping of the three cDNA libraries showed that, on average, 65.9% of clean sequences were mapped to the tobacco K326 reference genome (Table 2). These results demonstrated that the sequencing data were reliable and could be used for further analyzing differentially expressed genes associated with target traits.

**Table 1:** Statistical table of sequencing data of three materials.

Sample	Raw reads	Raw bases	Clean reads	Clean bases	Error rate (%)	Q20 (%)	Q30 (%)	GC content (%)
G70_1	83,981,574	12,681,217,674	83,075,818	12,324,430,722	0.0244	98.29	94.76	43.47
G70_2	89,563,688	13,524,116,888	88,790,214	13,068,587,523	0.0241	98.4	95.03	43.09
G70_3	94,593,544	14,283,625,144	93,595,804	13,764,269,216	0.0242	98.35	94.94	43.08
GDH11_1	74,585,534	11,262,415,634	74,033,572	10,963,664,867	0.0246	98.21	94.5	43.27
GDH11_2	83,101,872	12,548,382,672	82,620,736	12,250,716,518	0.0243	98.33	94.78	42.9
GDH11_3	90,796,556	13,710,279,956	90,038,852	13,299,062,270	0.0243	98.31	94.81	43.16
G70 × GDH11_1	96,273,928	14,537,363,128	95,551,060	14,099,671,149	0.0243	98.31	94.77	43.15
G70 × GDH11_2	92,506,634	13,968,501,734	91,988,384	13,556,441,950	0.0243	98.34	94.86	43.57
G70 × GDH11_3	75,007,222	11,326,090,522	74,555,956	11,064,020,575	0.0245	98.26	94.57	42.6

Raw reads: the total number of items of the original sequencing data (reads, representing the sequencing reading segment, one read is one); Raw bases: the total amount of raw sequencing data (i.e., the number of raw reads multiplied by the read length); Clean reads: total number of items of sequencing data after quality control; Clean bases: the total amount of sequencing data after quality control (i.e., the number of clean reads multiplied by the length of reads); Error rate (%): average error rate of sequencing base corresponding to quality control data; Q20 (%) and Q30 (%): evaluate the quality of sequencing data after quality control. Q20 and Q30, respectively, refer to the percentage of bases with sequencing quality of more than 99 and 99.9% in the total bases; GC content (%): the percentage of the sum of G and C bases corresponding to quality control data in the total base.

**Table 2:** Comparison results of root sequences on the reference genome.

Sample Names	Total reads	Total mapped	Multiple mapped	Uniquely mapped
G70_1	83,075,818	54,759,364 (65.91%)	1,166,278 (1.4%)	53,593,086 (64.51%)
G70_2	88,790,214	58,537,912 (65.93%)	1,492,868 (1.68%)	57,045,044 (64.25%)
G70_3	93,595,804	61,695,124 (65.92%)	1,529,132 (1.63%)	60,165,992 (64.28%)
GDH11_1	74,033,572	49,019,700 (66.21%)	1,021,789 (1.38%)	47,997,911 (64.83%)
GDH11_2	82,620,736	54,311,755 (65.74%)	1,455,276 (1.76%)	52,856,479 (63.97%)
GDH11_3	90,038,852	59,960,465 (66.59%)	1,735,791 (1.93%)	58,224,674 (64.67%)
G70 × GDH11_1	95,551,060	62,606,990 (65.52%)	1,345,318 (1.41%)	61,261,672 (64.11%)
G70 × GDH11_2	91,988,384	60,960,400 (66.27%)	1,395,315 (1.52%)	59,565,085 (64.75%)
G70 × GDH11_3	74,555,956	48,469,163 (65.01%)	1,364,515 (1.83%)	47,104,648 (63.18%)

Total reads: Statistics on the number of sequenced sequences after filtering (i.e., clean reads); Total mapped: Number of clean reads that can be located on the genome; Multiple mapped: The number of clean reads with multiple alignment positions on the reference sequence; Unique mapped: The number of clean reads that have unique alignment positions on the reference sequence.

**Figure 2:**
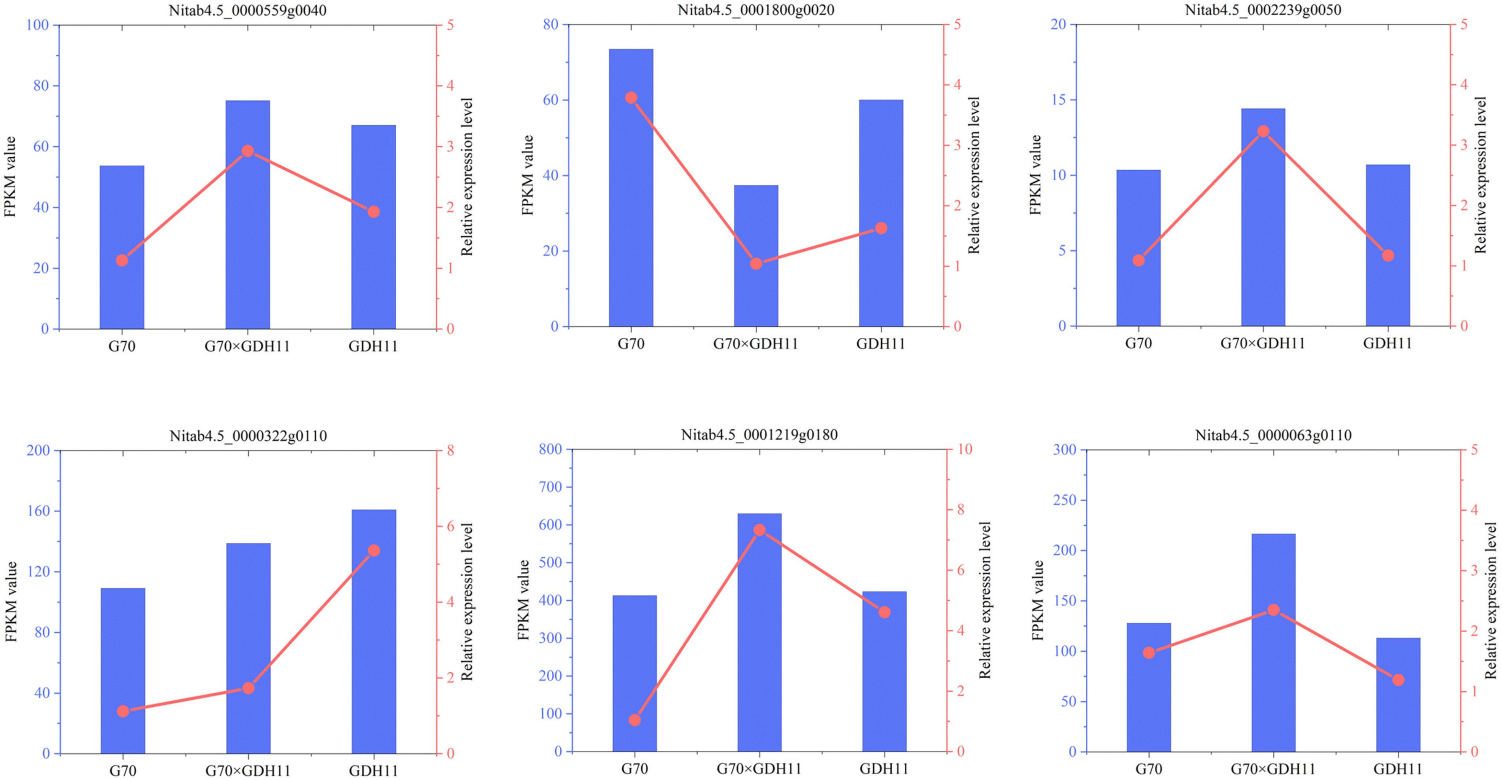
Validation of transcriptomics data through qRT-PCR analysis.

To evaluate the quality of the transcriptomics and gene differential expression level data, six genes were randomly selected for qRT-PCR analysis. The actin gene was used as an internal reference gene to analyze the level of expression of each gene. The genes and the corresponding primers used for qRT-PCR determination were listed in Supplementary Table S2. The changes in the expression of the selected genes based on the relative expression level followed a pattern similar to that found in the transcriptomics data (Figure 2), which indicated that the transcriptome profiling data were reliable. Therefore, we can carry out the next step of differential gene screening and expression analysis.

**Figure 3:**
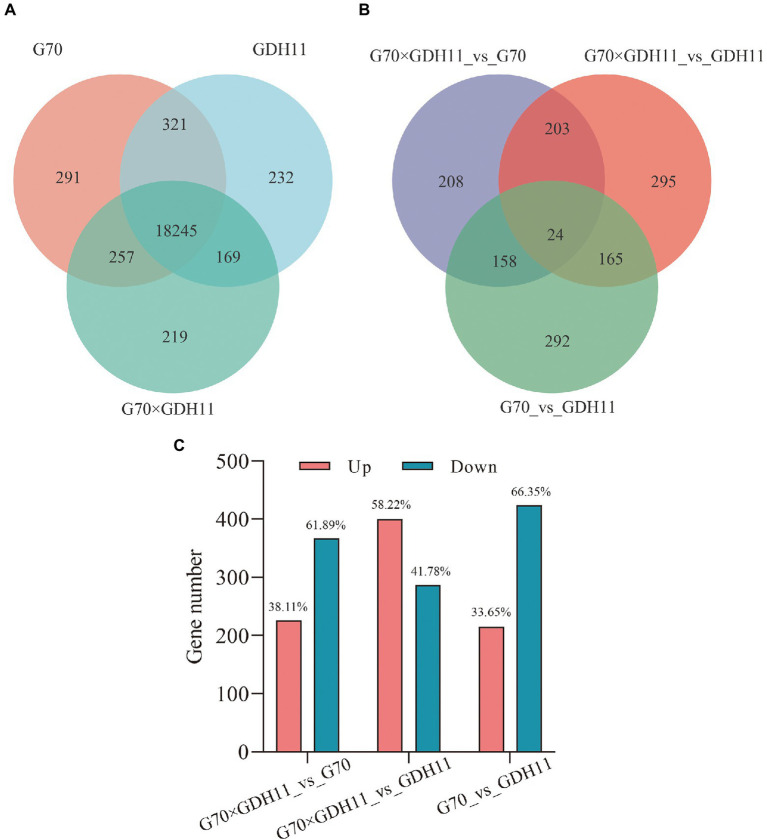
The transcriptional profiling statistics of different materials and comparison groups. **(A)** Venn diagram of the identified genes for the three tobacco varieties. **(B)** Venn diagram of the DEGs in the comparison groups G70_vs_GDH11, G70 × GDH11_vs_G70, and G70 × GDH11_vs_GDH11. **(C)** Statistics of up and down regulated differentiallyexpressed genes between hybrid and parental inbredlines.

To analyze the causes of tobacco heterosis at the transcriptional level, we compared the transcriptional information of the F_1_ hybrid with that of G70 and GDH11, as well as the transcriptional information between the parents. In the subsequent analysis, we considered 18,245 genes that were simultaneously identified in the three genotypes (Supplementary File 2, Figure 3A). Using the threshold of *p* ≤ 0.05 and |log2 Fold Change| ≥ 2, we identified 226 upregulated and 367 downregulated transcripts between the hybrid and G70. Similarly, 400 upregulated and 287 downregulated transcripts were observed between the hybrid and GDH11, and 215 upregulated and 424 downregulated transcripts were observed between G70 and GDH11 (Figure 3C). The results of a Venn diagram analysis showed that only 24 DEGs co-existed in the three groups (Figure 3B), which indicated that there were significant differences in the transcriptional ubiquitous expression of the three genotypes. Therefore, we speculated that these DEGs might be the reason that tobacco hybrids show heterosis in some traits.

### Identification and analysis of expression patterns of DEGs in the hybrid

**Figure 4:**
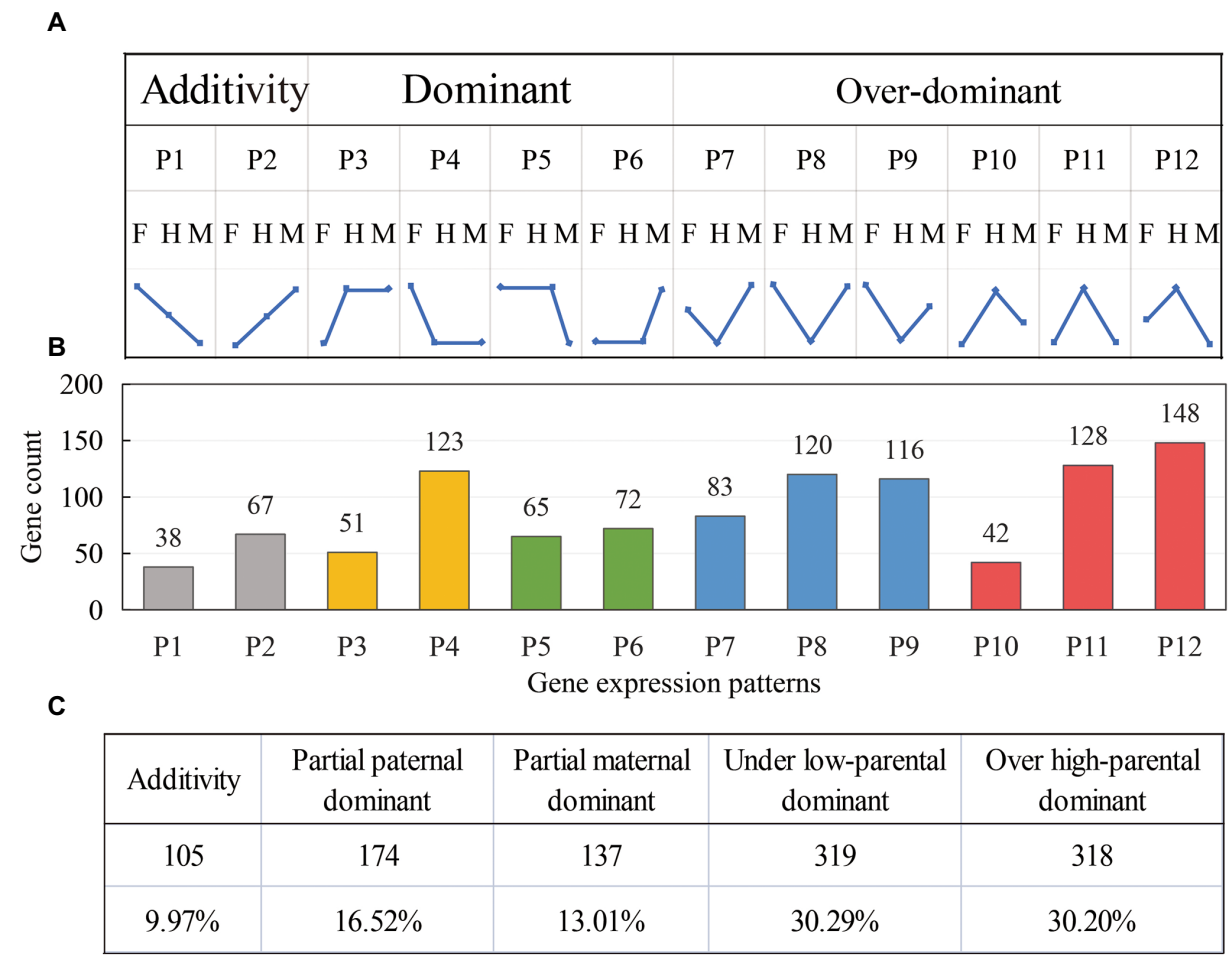
Identification and classification of differential gene expression patterns in the hybrids. **(A)** The division of 12 expression patterns; F: female parent, H: hybrid, and M: negative parent. **(B)** The distribution of DEGs in the hybrids among 12 expression patterns. **(C)** The number and proportion of DEGs in the five expression patterns.

To further analyze the effects of these DEGs in the formation of K^+^ content heterosis in tobacco leaves, we divided the differential genes into 12 expression patterns (P1–P12, Figure 4A) and determined the effect of additive and non-additive expression of the genes on heterosis of K^+^ content. Genes in the P1 and P2 patterns were additively expressed. The genes in the P3–P6 pattern showed dominant expression, in which P3 and P4 showed partial paternal dominant expression, and P5 and P6 showed partial maternal dominant expression. P7–P9 showed low parental dominant expression, and P10–P12 showed over-high parental dominant expression. We divided these 12 expression patterns into five categories (Figure 4B), and different colors were used to indicate an expression pattern type.

In the hybrid G70 × GDH11, only 9.97% (P1 and P2) of the gene expression levels were between the parents, showing an additive expression pattern (Figure 4C). The remaining 90.03% of the genes showed a non-additive expression pattern, which indicated that the formation of K^+^ content heterosis was more affected by the non-additive effect of the genes. Among the non-additively expressed genes, 29.53% (P3-P6) of the genes showed a dominant expression pattern, and 60.49% of the genes (P7–P12) showed an over-dominant expression pattern. The results indicated that the over-dominant expression pattern of DEGs played a major role in the formation of K^+^ content heterosis.

### Functional enrichment analysis of non-additively expressed genes

**Figure 5:**
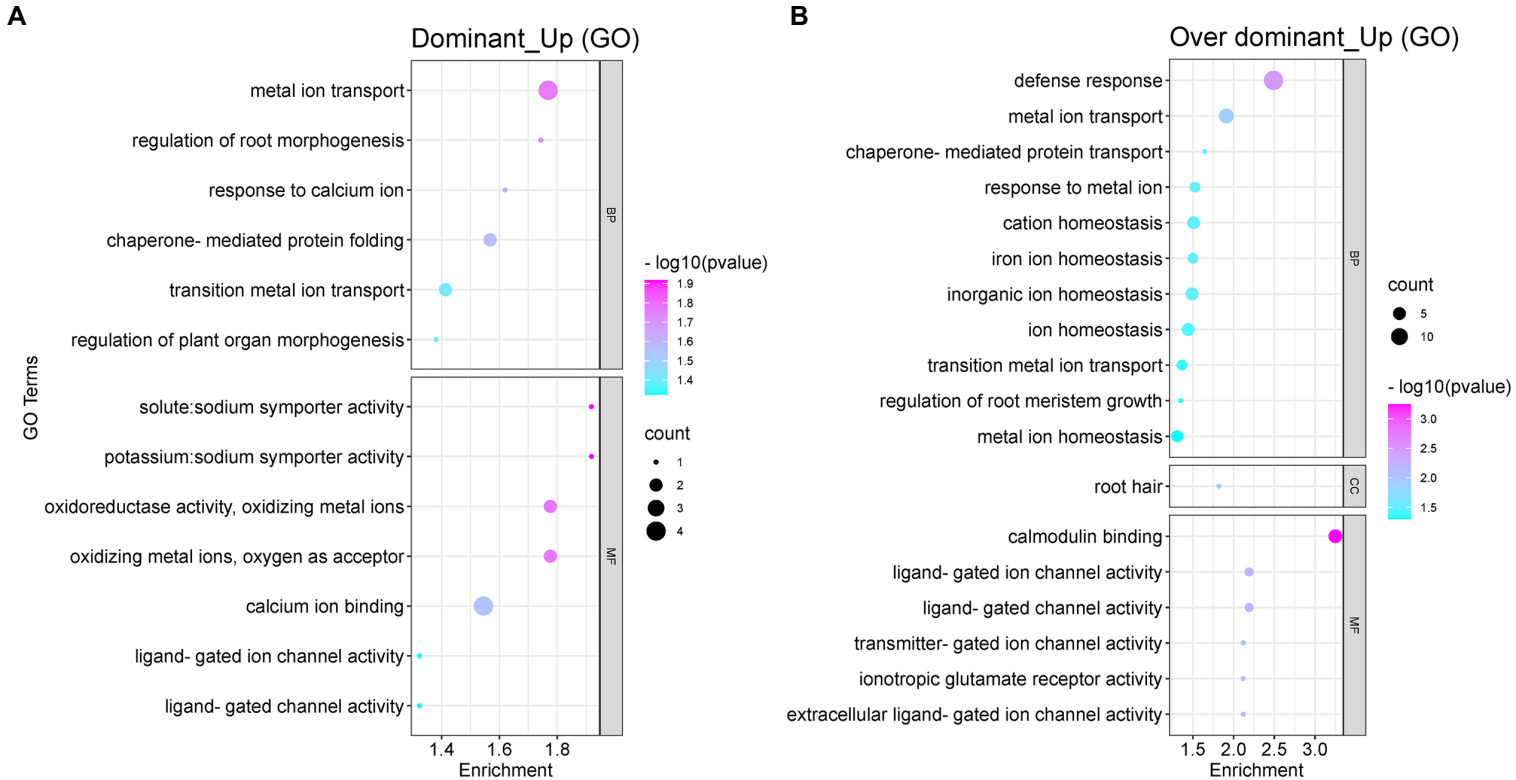
The GO functional enrichment analysis of the non-additive upregulated DEGs. **(A)** The GO functional enrichment analysis of the upregulated dominant DEGs. **(B)** The GO functional enrichment analysis of the upregulated over-dominant DEGs.

The degree of heterosis was found to depend on the non-additive effect of genes. Moreover, the non-additive expression of the genes affected the performance of hybrid offspring. To understand the biological functions of these non-additivity expressed genes, we performed the GO functional enrichment analysis on the gene sets with dominant (120 DEGs) and over-dominant upregulated (318 DEGs) expression. The GO enrichment analysis results of downregulated genes in non-additivity expression are shown in Supplementary Figures S1, S2. The results showed that the upregulated genes were mainly enriched in metal ion transport, calcium ion binding, potassium/sodium transporter activity, regulation of root morphogenesis, and ion channel activity (Figure 5A). The upregulated genes with over-dominant expression could be enriched in more GO functional terms, mainly including root defense response, metal ion transport and response, ion balance and homeostasis, ion channel activity, root meristem growth, and the regulation of root hair (Figure 5B). The results of the functional analysis indicated that these non-additively upregulated differential genes in the hybrid G70 × GDH11 were good sources of potassium uptake in the hybrid. Their overexpression facilitates root growth and regulation of the meristem. These genes are also involved in ion transport and the regulation of ion channel activity, which enables K^+^ in the soil to be more efficiently absorbed by the roots and transported to the tobacco leaves, thus causing the potassium content in the leaves of the hybrids to show heterosis.

**Figure 6:**
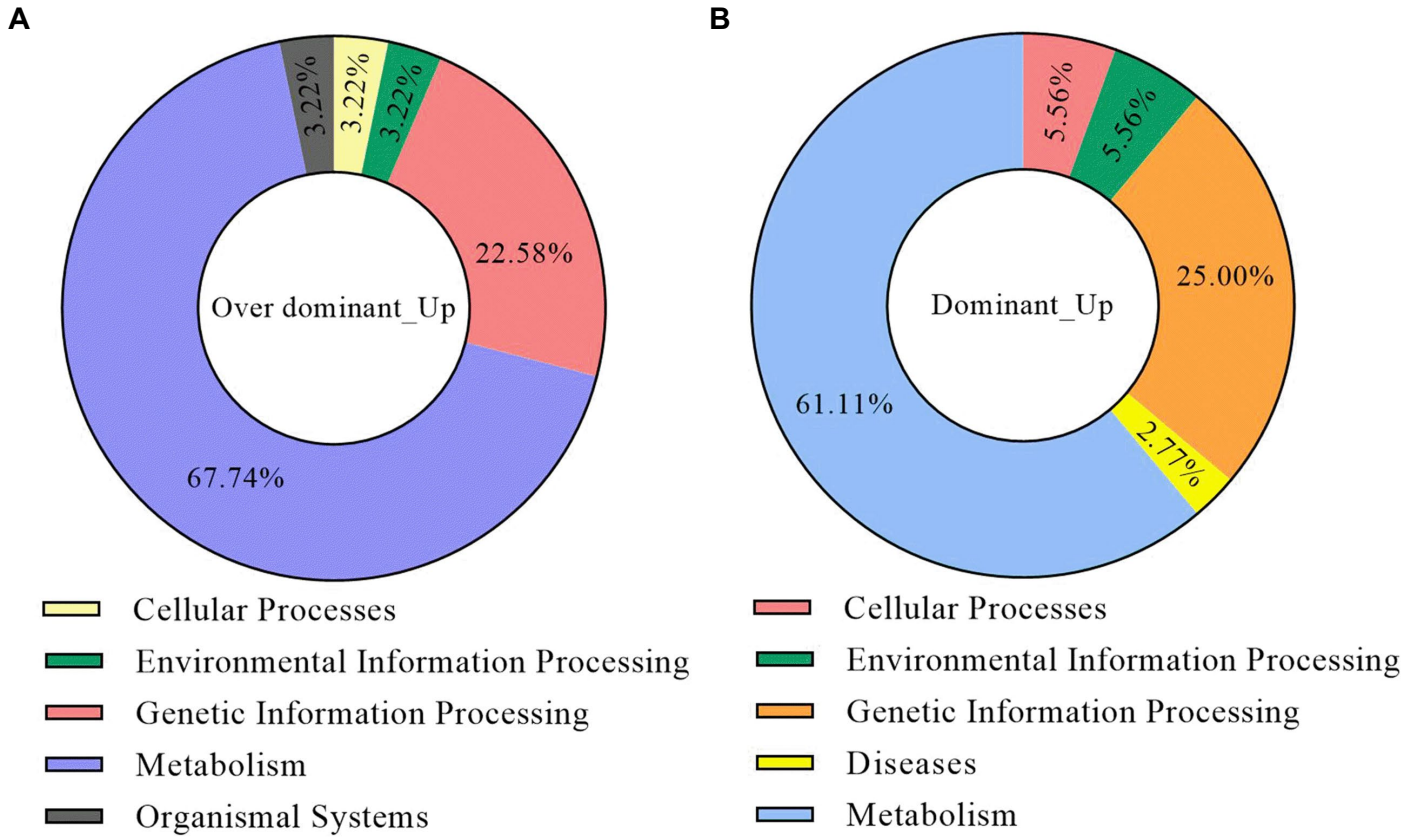
The KEGG pathway enrichment analysis of the non-additive upregulated DEGs. **(A)** The classification of the KEGG enrichment results for dominant upregulated DEGs. **(B)** The classification of the KEGG enrichment results for the over-dominant upregulated DEGs.

To understand the biological pathways associated with the non-additive upregulated DEGs, we performed the KEGG pathway enrichment analysis for these genes. The results showed that the DEGs of the dominant and over-dominant gene sets were enriched in 36 and 62 pathways, respectively (Supplementary Files 3, 4). The first-level classification results of these pathways showed that most of them belonged to the categories of metabolism, genetic information processing, cellular processes, and environmental information processing (Figures 6A,B). The non-additive upregulated DEGs were mainly involved in energy metabolism, starch and sucrose metabolism, photosynthesis, carbohydrate formation, amino acid metabolism, plant hormone signal transduction, and signaling pathway, indicating that the significant expression of these biological processes in hybrids provides energy for the development of the roots and the absorption and transport of K^+^, which is helpful to the accumulation of K^+^ and the production of K^+^ content superiority in hybrids.

### Mining of key genes regulating potassium absorption in hybrids

**Figure 7:**
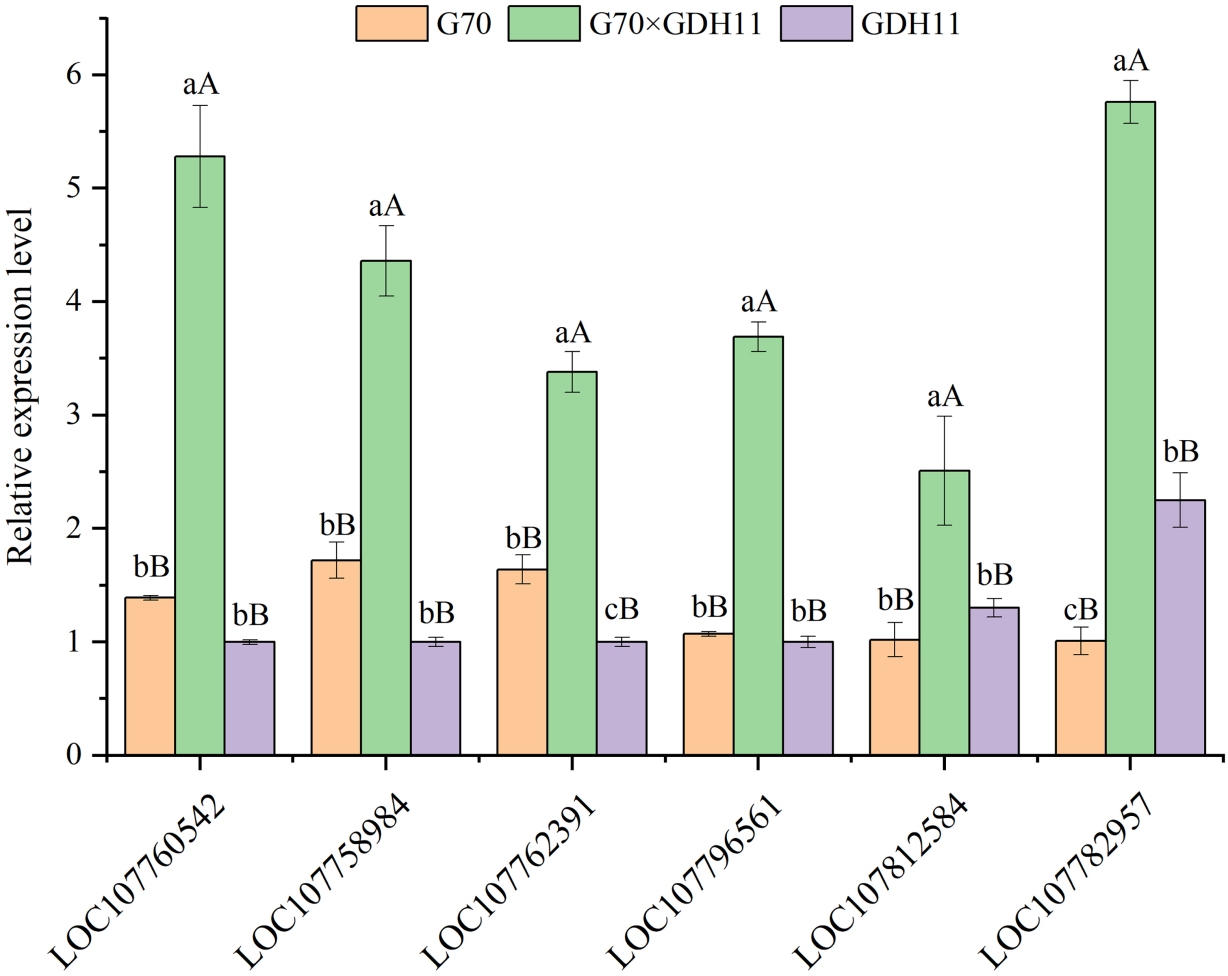
Expression level analysis of K^+^ related genes by qRT-PCR. The lowercase alphabets represent a significant difference (*p* < 0.05), while the uppercase alphabets represent a highly significant difference (*p* < 0.01).

To further analyze the basis of the formation of tobacco K^+^ heterosis, we performed homology alignment and annotation analysis of the genes associated with the important GO entries (Table 3). The results showed that the probable potassium transporter 17 (*NtKT17*) and potassium transporter 5-like (*NtKT5*), associated with potassium transport, showed over-dominant upregulation and dominant expression in the hybrid G70 × GDH11, respectively. They play an important role in metal ion transport in plants. The glutamate receptor 2.2-like and glutamate receptor 2.8-like associated with ligand-gated channel activity showed an over-dominant upregulation expression in the hybrids. Some calcium-dependent protein kinase 28-like and calmodulin-binding protein 60 D-like related to the potassium ion absorption mechanism also showed a over-dominant upregulation. Furthermore, tabacum protein detoxification 42-like, involved in root hair development, and LOC107782957, involved in root morphology regulation, also showed over-dominant and dominant upregulation expression in the hybrids, respectively. We selected six genes related to K+ absorption and transport for the Real-Time Fluorescence Quantitative PCR detection (Figure 7). The genes and the corresponding primers are listed in Supplementary Table S3. The results showed that the expression level of these genes in hybrid G70 × GDH11 was significantly higher than that in two parental inbred lines, which indicated that these genes indeed have significant over-dominant and dominant expression patterns in hybrids. These results suggested that the over-dominant and dominant upregulation of key genes associated with K^+^ uptake and transport leads to the generation of K^+^ heterosis.

**Table 3:** Statistics of genes related to potassium content heterosis.

Pattern	Gene ID	Description	F_1_	MP	FC	H_FPKM
Over-dominant	LOC107784880	Laccase-12-like	17.60	8.10	2.17	117.24%
	LOC107779045	Sodium/hydrogen exchanger 3-like	0.21	0.10	2.03	103.23%
	LOC107787287	Monocopper oxidase-like protein SKU5	1.34	0.94	1.43	42.96%
	LOC107760542	Probable potassium transporter 17	12.60	3.74	3.37	236.99%
	LOC107759178	Solute carrier family 40 member 3	124.56	65.82	1.89	89.26%
	LOC107758984	Tabacum protein detoxification 42-like	4.83	2.82	1.71	71.39%
	LOC107795534	Triosephosphate isomerase	22.25	12.21	1.82	82.28%
	LOC107783543	Oxalate–CoA ligase-like	1.23	0.83	1.49	48.59%
	LOC107791078	Calcium uniporter protein 5	4.01	1.96	2.05	104.94%
	LOC107790202	Probable glutamate carboxypeptidase 2	1.10	0.54	2.02	102.45%
	LOC107762391	Glutamate receptor 2.2-like	7.86	5.48	1.44	43.58%
	LOC107796561	Glutamate receptor 2.8-like	1.51	0.84	1.79	79.37%
	LOC107810988	Calmodulin-binding protein 60 D-like	10.83	7.22	1.50	50.03%
	LOC107769099	MLO-like protein 6	1.33	0.73	1.82	82.19%
	LOC107800917	Calcium-transporting ATPase 2	6.17	3.25	1.90	89.74%
	LOC107771176	Calmodulin-binding protein 60 B-like	0.12	0.08	1.64	64.44%
	LOC107765993	Uncharacterized LOC107765993	56.00	19.93	2.81	180.95%
	LOC107825481	Calcium-dependent protein kinase 28-like	5.39	2.21	2.45	144.60%
Dominant	LOC107812584	Potassium transporter 5-like	6.55	2.96	2.21	121.05%
	LOC107777935	L-ascorbate oxidase homolog	99.27	50.36	1.97	97.15%
	LOC107792406	L-ascorbate oxidase homolog	1.15	0.46	2.49	149.10%
	LOC107782957	Uncharacterized LOC107782957	0.74	0.28	2.64	163.91%
	LOC107803414	Heat shock cognate 70 kDa protein 1-like	77.18	38.92	1.98	98.30%
	LOC107809995	Calcineurin subunit B-like	0.51	0.32	1.59	58.55%
	LOC107769668	Alpha-amylase-like	2.39	1.84	1.30	29.84%
	LOC107793486	Uncharacterized LOC107793486	21.86	13.28	1.65	64.65%
	LOC107783728	Peroxygenase-like	6.09	4.32	1.41	41.16%
	LOC107800970	Uncharacterized LOC107800970	51.60	18.09	2.85	185.21%

MP represents the average value of gene expression of two parents; FC represents the foldchange of hybrid (G70 × GDH11), and MP. H_ MP represents the heterosis value of gene expression.

## Discussion

The growth and development of plants are inseparable from the action of various essential elements. Among them, K^+^ is a major element that affects various aspects of plant growth (Sano et al., 2009). Especially for food crops and cash crops, an increase in the K^+^ content can often directly improve the yield and quality (Yang and Wang, 2017). Thus, the absorption and transport of K^+^ have gained attention in recent years (Pettigrew, 2008). The quality of the tobacco plant is affected by K^+^. However, studies on K^+^ channels and transporters in tobacco are limited, especially on the genes regulating K^+^ heterosis. Our study showed that the heterosis expression of K^+^ content in tobacco was prominent and affected by the non-additive effect of the genes, of which the over-dominant effect played a major role. The results showed that breeding new tobacco varieties with high K^+^ content through heterosis is feasible.

With the development of modern molecular biology and advancement in quantitative genetics, research on heterosis has progressed. Transcriptomics is commonly used to analyze the mechanism of crop heterosis formation and satisfactory results have also been achieved in many studies (Palareti et al., 2016; Jiang et al., 2017; Birdseye et al., 2021; Liu et al., 2021, 2022). A correlation between heterosis and gene expression levels was shown in many studies (Wei et al., 2009; He et al., 2010). However, studies on the heterosis of important traits in tobacco are limited. Our transcriptomics results revealed that 35,520 genes were transcribed in at least one genotype (Supplementary File 5), which showed the active genes that regulate K^+^ uptake and transport in tobacco.

Potassium ion is absorbed from the soil by root epidermal cells and cortical cells in plants, and ion channels and transport carriers play an important role in this process (Sánchez-Caldero et al., 2009). Liu et al. (2019) showed that the expression of *NtTPK1* and *NtORK1* might be related to the K^+^ content and the formation of heterosis. Previous studies found that the influx K^+^ channel genes *AKT1*, *NtKC1*, *KAT1*, and K^+^ transporter gene *NtTPK1* showed dominant and over-dominant expression in the roots and leaves of the hybrids, indicating that these genes played a key role in the formation of K^+^ content heterosis in hybrid tobacco leaves (Luo et al., 2022). Some studies have shown that the K+ transporter plays a major role in absorbing K^+^ from the rhizosphere and can also be used as a low-affinity K^+^ transporter at high K^+^ concentrations (Kim et al., 1998; Ahn et al., 2004). In this study, probable potassium transporter 17 (*NtKT17*) and potassium transporter 5-like (*NtKT5*) showed upregulated over-dominant and dominant expression in the hybrids, respectively, with advantages in expression. The results further showed that these two genes were also involved in the absorption of K^+^ in the roots, and the absorption rate in the hybrids was higher than that in the parents.

By opening and closing, ion channels regulate the speed of movement of specific substances in and out of cells, required for various cell functions (Hedrich, 2012). K^+^ channel parenchyma is a transmembrane protein that is present on the cytoplasmic membrane or the inner membrane of plant cells (Hosy et al., 2003; Dreyer and Uozumi, 2011). Among these, ligand-gated channels, also known as chemical-gated ion channels, open after certain transmitters bind to the channel protein receptor molecule, thus allowing Na^+^, Ca^2+^, or K^+^ to pass through (Liu et al., 2006a; Xie et al., 2020). We found that the glutamate receptor 2.2-like and glutamate receptor 2.8-like associated with ligand-gated channel activity showed an upregulated over-dominant expression in the hybrid. Therefore, we speculated that these two types of glutamate receptors might also play an important role in the formation of heterosis in tobacco K^+^ content, thus contributing to K^+^ transport by the roots. However, their mechanism of action needs to be further analyzed and confirmed.

Plant root architecture plays an important role in nutrient absorption, and there are considerable differences in root architecture between different species of plants or different varieties of the same plant species (Hao, 2015). Studies showed that the number of roots was positively correlated with the K^+^ content of leaves in cotton (Hsu et al., 1978). Root hairs can absorb nutrients in the soil more effectively by increasing the contact area with the soil (Jungk, 2001; Wang et al., 2016). From the perspective of molecular biology, ion channels and transporters in root hairs greatly facilitate nutrient absorption (Libault et al., 2010). Our results showed that the uncharacterized LOC107782957 gene involved in the regulation of tobacco root morphogenesis upregulated dominant expression in the hybrids. Furthermore, protein detoxification 42-like, involved in root hair development, and probable glutamate carboxypeptidase 2, involved in the regulation of root meristem growth, showed an upregulated over-dominant expression pattern. The results indicated that the upregulated expression of these key genes in the hybrids promotes root growth and development, which is crucial for nutrient uptake by the roots. Many researchers argue that energy metabolism strongly affects the growth and development of plant roots (Yokota et al., 2003; Libault et al., 2010; Wang et al., 2016). Similar to the results of those studies, the KEGG enrichment results of this study also indicated that many upregulated non-additive DEGs are involved in energy metabolism and carbohydrate formation, thus enhancing the root absorption capacity of tobacco hybrids.

Crop heterosis formation is an extremely complex process. Our study provided a new perspective on the mechanism of K^+^ content heterosis at the transcriptional level and might provide new information for cultivating tobacco varieties with high K^+^ content. Potassium exists in plants in an ionic state, and its accumulation is jointly influenced by the growth and development of the root, the expression of K^+^ uptake genes and transporters, K^+^ channel activity, and the homeostasis of ions. Overexpression of the genes associated with these physiological functions in hybrids contributes to K^+^ absorption and accumulation, thus exhibiting K^+^ content heterosis.

## Data availability statement

The data presented in the study are deposited in the NCBI Sequence Read Archive (SRA) repository, accession number PRJNA836042.

## Author contributions

ZM, WL, and RL conceived and designed the research. WL, PW, and YK conducted field experiments. KP, SZ, and TL conducted the molecular biology experiments. ZM, LD, and RJ analyzed the data. ZM, KP, and YH wrote the manuscript. ZM and RL revised the manuscript. All authors contributed to the article and approved the submitted version.

## Funding

This research was funded by the National Science Foundation of China (no. 32060510), the Talent Cultivation Project for High-Level Renovation Plan of Guizhou Province (no. [2016]5663), the Guizhou Provincial Key Foundation (no. [2019]1405), and Tobacco Company of Guizhou Province (nos. 2020XM07 and 201904).

## Conflict of interest

The authors declare that the research was conducted in the absence of any commercial or financial relationships that could be construed as a potential conflict of interest.

## Publisher’s note

All claims expressed in this article are solely those of the authors and do not necessarily represent those of their affiliated organizations, or those of the publisher, the editors and the reviewers. Any product that may be evaluated in this article, or claim that may be made by its manufacturer, is not guaranteed or endorsed by the publisher.

## Footnotes

^1^ https://solgenomics.net/organism/Nicotiana_tabacum/genome

## Supplementary material

Supplementary File 1 The original data of Figure 1.

Supplementary File 2 The number of genes and information on gene expression identified for the three genotype materials.

Supplementary File 3 The KEGG annotation results of the dominant upregulated DEGs.

Supplementary File 4 The KEGG annotation results of the over-dominant upregulated DEGs.

Supplementary Figure S1 The GO functional enrichment analysis of the downregulated dominant DEGs.

Supplementary Figure S2 The GO functional enrichment analysis of the downregulated over-dominant DEGs.
