# Supplementary figures and images for: Comparative transcriptome analysis between inbred lines and hybrids provides molecular insights into K+ content heterosis of tobacco (Nicotiana tabacum L.)
Source: Front Plant Sci. 2022 Aug 5;13:940787. doi: 10.3389/fpls.2022.940787 (PMC9389268; doi:10.3389/fpls.2022.940787)

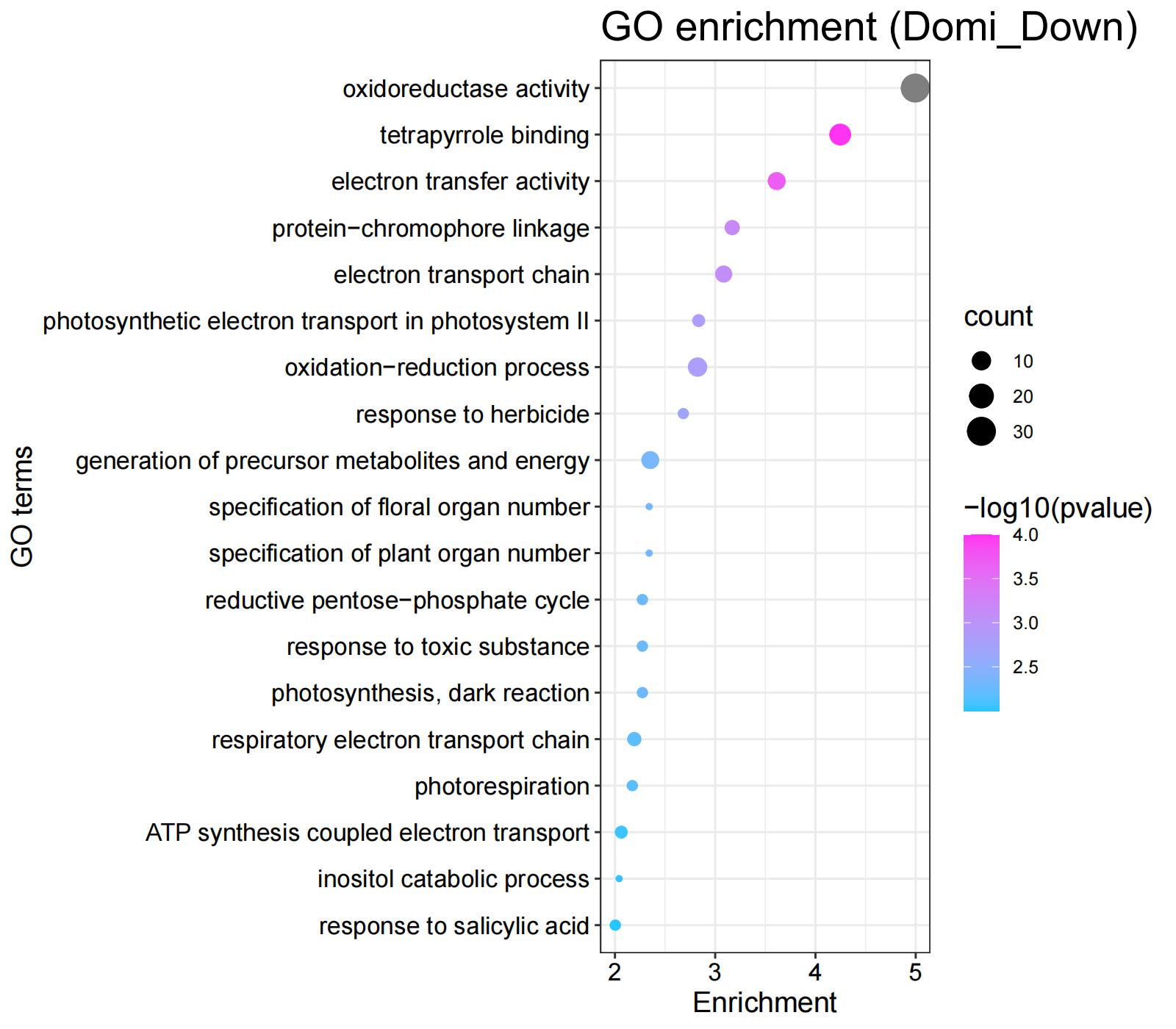

Supplement: Supplementary Figure S1 — The GO functional enrichment analysis of the downregulated dominant DEGs. [file Image_1.jpeg]

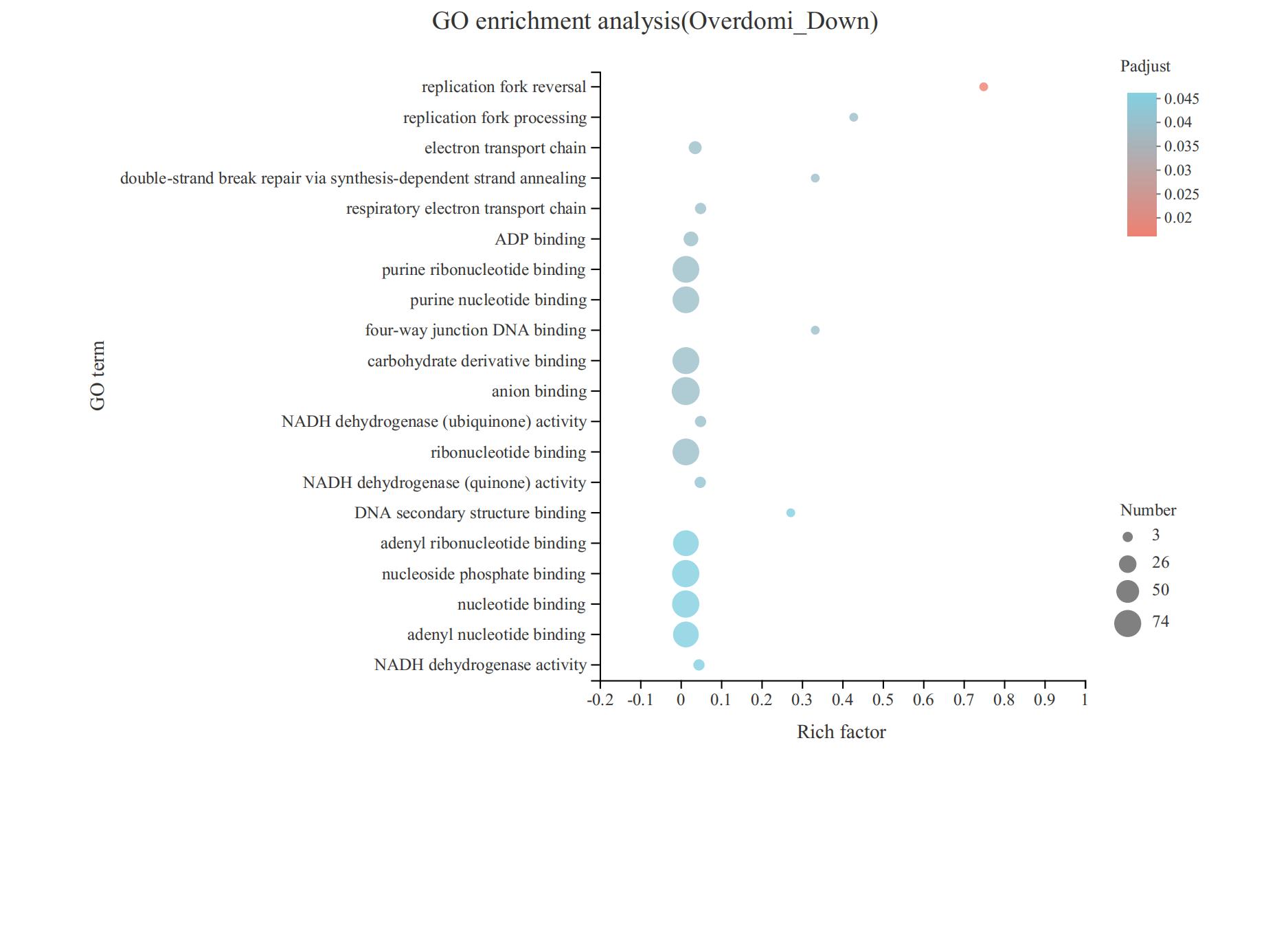

Supplement: Supplementary Figure S2 — The GO functional enrichment analysis of the downregulated over-dominant DEGs. [file Image_2.jpeg]
