# Supplementary material for: Comparative transcriptome analysis between inbred lines and hybrids provides molecular insights into K+ content heterosis of tobacco (Nicotiana tabacum L.)
Source: Front Plant Sci. 2022 Aug 5;13:940787. doi: 10.3389/fpls.2022.940787 (PMC9389268; doi:10.3389/fpls.2022.940787)
Supplement: Supplementary file 7 [file Data_Sheet_5.docx]

| **TABLE S1│** Performance of agronomic traits of three genotypic materials | | | | |
| --- | --- | --- | --- | --- |
| Traits  Materials | Stem girth  (cm) | Leaf length  (cm) | Leaf width  (cm) | Dry weight  (g) |
| G70 | 8.05 abA | 58.45 bB | 22.88 cB | 48.34 bA |
| GDH11 | 7.69 bA | 57.54 bB | 23.61 bAB | 48.15 bA |
| G70×GDH11 | 8.91 abA | 66.19 aA | 24.42 aA | 52.36 aA |
| Notes: Significant differences among the agronomic characters at P < 0.05 and P < 0.01 were determined using the Duncan’s new multiple range test. The lowercase alphabets represent a significant difference (*P* < 0.05); uppercase alphabets represent an extremely significant difference (*P* < 0.01). | | | | |

| **TABLE S2│** Primer sequences of genes used in real-time qPCR analysis for transcriptome result validation | | |
| --- | --- | --- |
| Accession | Primer sequence | Reverse primer |
| Actin | GTGTTATGGTCGGAATGGG | TGGAACGCTAAATGTCTCAAA |
| Nitab4.5_0000559g0040 | ACATGAAGGTAAAACTACAGAGCTA | GCTGGACAAGTCATGCAAAC |
| Nitab4.5_0001800g0020 | AGGCAAGTTTGAACTCAGTGG | AGTCGACCCTTGTCGTTGGT |
| Nitab4.5_0002239g0050 | AAGTGAAGGAGGAGGGAGTGA | GTCTAGCATCTGGACCAACCAA |
| Nitab4.5_0000322g0110 | GATTCTACCACCGCTTCCGC | CTTCCCTCTCGCACGTGAAC |
| Nitab4.5_0001219g0180 | AGATGGTGGCATCCCTGTTG | CTTGTCTCCTGGCACGCTTA |
| Nitab4.5_0001219g0180 | CTTCTGCTGACATCTCGGGG | AATAGCAAGGGCAGGAGCAC |
| Nitab4.5_0000063g0110 | GGCTGCAATGTGACAGGAATG | CGTCCATTGTTCCCTTCACT |

**TABLE S3│** Primer sequences of genes used in real-time qPCR analysis for expression verification of K^+^ related genes

| Accession | Primer sequence | Reverse primer |
| --- | --- | --- |
| Actin | GTGTTATGGTCGGAATGGG | TGGAACGCTAAATGTCTCAAA |
| LOC107760542 | CACCTGCCATCTCAGTGTTGT | CCAACGAGAGGAGTGGTAAGAG |
| LOC107758984 | CATTGGCTGCACGGTTAGGA | TGCTGCGACGAACGGAATAC |
| LOC107762391 | CTGGCGGTCTGGATACTTGAAC | GCTCGCTAACATCTGTGATTGC |
| LOC107796561 | CATCCGCTGCCATATACTTGCT | AGGAAGTGCTGCTCTGATGAAG |
| LOC107812584 | AACCAGGAGCCAGAGGACAG | CACAACCGCATCTTCGCCTAA |
| LOC107782957 | GAGCCTCCAACTCCACATTCAA | CGAGTGAGCCAGAACGAAGTAA |
